# The Immunobiology of Prostanoid Receptor Signaling in Connecting Innate and Adaptive Immunity

**DOI:** 10.1155/2013/683405

**Published:** 2013-08-20

**Authors:** Hedi Harizi

**Affiliations:** Faculty of Dental Medicine, University of Monastir, 5019 Monastir, Tunisia

## Abstract

Prostanoids, including prostaglandins (PGs), thromboxanes (TXs), and prostacyclins, are synthesized from arachidonic acid (AA) by the action of Cyclooxygenase (COX) enzymes. They are bioactive inflammatory lipid mediators that play a key role in immunity and immunopathology. Prostanoids exert their effects on immune and inflammatory cells by binding to membrane receptors that are widely expressed throughout the immune system and act at multiple levels in innate and adaptive immunity. The immunoregulatory role of prostanoids results from their ability to regulate cell-cell interaction, antigen presentation, cytokine production, cytokine receptor expression, differentiation, survival, apoptosis, cell-surface molecule levels, and cell migration in both autocrine and paracrine manners. By acting on immune cells of both systems, prostanoids and their receptors have great impact on immune regulation and play a pivotal role in connecting innate and adaptive immunity. This paper focuses on the immunobiology of prostanoid receptor signaling because of their potential clinical relevance for various disorders including inflammation, autoimmunity, and tumorigenesis. We mainly discuss the effects of major COX metabolites, PGD2, PGE2, their signaling during dendritic cell (DC)-natural killer (NK) reciprocal crosstalk, DC-T cell interaction, and subsequent consequences on determining crucial aspects of innate and adaptive immunity in normal and pathological settings.

## 1. Introduction

Prostanoids are biologically active molecules that have various and potent effects on almost all cells and tissues in physiological and pathophysiological conditions [1]. These fascinating molecules can sustain homeostatic functions and mediate pathogenic mechanisms, including tumorigenesis autoimmunity, cardiovascular diseases, and inflammation [2]. Known as potent inflammatory lipid mediators, prostanoids may function in both the promotion and resolution of the inflammatory response [3]. Their biosynthesis is suppressed by nonsteroidal anti-inflammatory drugs (NSAIDs) that are clinically relevant molecules widely consumed as analgesics or antipyretics drugs. The use of NSAIDs as anti-inflammatory agents highlights the proinflammatory role of prostanoids. For example, NSAIDs reduce fever caused by infectious, inflammatory, or neoplastic diseases, by inhibiting the synthesis of PGE2 [4]. Moreover, epidemiological studies have provided evidence that NSAIDs that inhibit PG synthesis by acting on COX enzymes can significantly reduce the risk of cancer development [5], suggesting that prostanoids may play a key role in tumorigenesis. In addition, defect in antitumor immunity has been reported to be associated with increased expression of COX-2-derived PGE2 [6–8].

Cell activation by growth factors, inflammatory stimuli, or mechanical trauma, resulted in the induction of prostanoid synthesis [9]. Rapid recognition of microbial lipopolysaccharide (LPS) by toll-like receptor-4 (TLR-4) is an important pathway, which provides an ideal model for the activation of prostanoid production and signaling [10]. Several lines of evidence showed that LPS of gram-negative bacteria induces the expression and activity of cytosolic phospholipase A2 (cPLA2), which catalyzes the release of endogenous AA from the cell membrane [11, 12]. The expression and activity of cPLA2 have been documented in various cell types, such as human leukocytes and murine dendritic cells (DCs) [13–15]. AA released from cell membrane by the action of cPLA2 can be converted by COX enzymes into the unstable cyclic endoperoxides PGG2 and PGH2. Specific downstream isomerases and synthases are involved in the biosynthesis of many prostanoids, including TXs, PGs, and prostacyclins [16]. 

COX enzymes exist as two distinct isoforms, *COX-1* and *COX-2*. *COX-1* is a housekeeping gene expressed constitutively in most cells and involved in physiological processes, such as gastric epithelial cytoprotection and homeostasis. *COX-2* is usually absent under basal conditions but can be induced by several stimuli, such as cytokines and other inflammatory factors. COX-2 enzyme can be induced by bacterial LPS mimicking bacterial infection [17]. Physiological activation of CD40-CD40 Ligand pathway, which occurs during antigen presentation by DCs, can induce the expression of COX-2 enzyme and the production of proinflammatory PGs [18]. CD40-induced production of some prostanoids has also been found in other cell types, such as fibroblasts [19], endothelial cells [20], and monocytes [21].

COX-2 pathway is an important source of prostanoid formation in inflamed tissue and cancer [22, 23]. There is emerging evidence that COX-2-derived prostanoids, mainly PGD2 and PGE2, play a crucial role in the function of all the components of the immune system [24]. In addition, the crosstalk between immune cells that influences subsequent adaptive immune responses can be modulated by PGD2 and PGE2 receptor signaling. Given the potent immunomodulatory effects of PGD2 and PGE2 [25–27], it is not surprising that cells that produced large amounts of these lipid mediators are considered to be the most powerful modulators of inflammatory processes and immune function. For these reasons, understanding how prostanoid receptor signaling affect immune cell crosstalk and functions may be exploited for rational development of immunotherapeutic strategies in various diseases ranging from inflammation and allergic diseases to cancer. Investigating the role of prostanoids in connecting innate and adaptive immunity is very complex because of differential profile of prostanoid biosynthesis within immune cells, multiple prostanoid-synthesizing enzymes, and different receptor signaling pathways with sometimes opposite effects. Moreover, epigenetic modifications in prostanoid genes represent additional levels of complexity for understanding immunological processes involved in normal and pathological settings.

## 2. Differential Profile of Prostanoid Biosynthesis in Immune Cells 

Depending on the prostanoid type, activated signaling pathway, and cellular target, prostanoids exert different and sometimes opposite actions [28]. These lipid mediators are distributed in all tissues and the profile of prostanoid production depends on the cell type and the differential expression and distribution of enzymes involved in their biosynthetic pathways within cells involved in generating inflammatory response. For example, mast cells that are resident immune cells, mainly located in the lung, gut, and skin, are known to be major producer of PGD2 when activated in response to allergen exposure [29, 30]. Other cell types, such as platelets, alveolar macrophages, Th2 cells, and osteoblasts, can also express PGD2-synthesizing enzymes and produce PGD2, albeit at much lower levels [31]. 

Among immune and inflammatory cells, macrophages have been reported to be an important source of AA-derived metabolites [32]. They are able to produce various lipid mediators, especially TXA2 and PGE2, the most abundant PGs produced in the body [33]. The profile of prostanoid production by macrophages can be altered upon cellular activation. While resting macrophages produce TXA2 in excess of PGE2, this ratio changes to favor PGE2 production in response to LPS activation [33]. Other cell types, such as fibroblasts, endothelial cells, and some types of malignant cells can produce PGE2. Human monocyte-derived DCs and bone marrow-derived DCs, the most potent antigen presenting cells (APC) of the immune system, produced high levels of PGE2, but not PGD2, as we and others have previously reported [34–36]. In another study, it has been reported that skin DCs express hematopoietic PGD synthase and also function as a source of PGD2, which can be nonenzymatically converted to different physiological metabolites, particularly 15-deoxy-Δ^12,14^ prostaglandin J2 (15d-PGJ2), known as a potent anti-inflammatory factor [37, 38].

The general consensus is that cells of the innate immunity, such as tissue macrophages and sentinel DCs, are major contributors of local prostanoids [39]. However, cells of adaptive immune response have been reported to be unable to synthesize prostanoids. The only exception is adaptive regulatory T cells as they express COX-2 enzyme and synthesize high levels of PGE2 [40]. Generated by immune or nonimmune cells, prostanoids can act locally in an autocrine and paracrine fashion and affect the function of neighboring cells. It has been reported that the endogenously released prostanoids represent an important mechanism by which APCs, including DCs and macrophages, regulate their own function [41, 42] and the function of other cell types, such as T, B, and NK cells [43, 44]. Although some cells of innate and adaptive immunity appeared to be unable to synthesize prostanoids, such as PGE2 and PGD2, they are described as powerful prostanoid-responding cells, as they express various prostanoid receptors. 

## 3. Prostanoid Receptor Expression and Signaling 

Studies on AA-derived lipid mediators showed that cells of the immune system are both sensitive to and a source of inflammatory prostanoids [1]. These lipid mediators exert their autocrine and/or paracrine functions through various receptor types with different location and signaling pathways [45]. Given the evanescent nature of some prostanoids, especially thromboxane and prostacyclin that have half-lives on the order of seconds to a few minutes, these compounds must act near their sites of synthesis. 

The major effects of prostanoid lipid mediators are mediated through specific G-protein-coupled cell membrane receptors that differ in their expression and intracellular signaling pathways [46]. PGE2, one of the best known and most well-studied prostanoids, exerts its effects through four distinct receptor subtypes (EP1–EP4). Two PGD2 receptors termed DP1 and DP2 bind PGD2 and mediate its effects on target cells [47, 48]. The receptors that bind PGF2*α*, PGI2, and TxA2 are FP, IP, and TP, respectively. EP2, EP4, IP, and DP1 signal through Gs-mediated increases in intracellular cyclic adenosine monophosphate (cAMP). However, EP1, FP, and TP activate phosphatidylinositol metabolism, leading to the formation of inositol trisphosphate with mobilization of intracellular Ca^2+^ stores. The PGD2 receptor DP2 also termed CRTH2 (chemoattractant receptor-homologous molecule) is expressed on Th2 lymphocytes [49] and is coupled with G_*α*i_-type G protein [47].

Although the major effects of COX-derived lipid mediators are mediated through cell surface receptors, some prostanoids and their metabolites may act on target cells through nuclear receptors located at the nuclear envelope [50]. The most important example is 15d-PGJ2, the physiological bioactive metabolite of PGD2, and the natural ligand for the Peroxisome proliferator-activated receptor-*γ* (PPAR*γ*) [51], which is a member of the nuclear receptor superfamily of ligand-dependent transcription factors. 15d-PGJ2 has garnered much interest because despite of its role in cytoprotection and inhibition of cellular proliferation [52], it possesses potent anti-inflammatory properties by inhibiting the transcription of pro-inflammatory mRNAs [53].

It has been reported that cellular components of the immune system express various prostanoid receptors [33]. The distribution of prostanoid receptors on immune cells differs from the distribution of prostanoid-specific synthases. While prostanoid receptors are expressed on cells of both innate (APC) and adaptive (T and B lymphocytes) immune systems, prostanoid synthases, on the other hand, are expressed mainly by the cells of inflammation (phagocytes) but not on T and B lymphocytes. Thus, stimulated cells of innate immunity, at a site of inflammation, will produce prostanoids, which in turn, will modulate their function in an autocrine fashion. Prostanoids will also contribute, with other inflammatory mediators, to the regulation of adaptive immunity through the innate system. These data clearly demonstrated that by their paracrine signaling, prostanoid receptors play critical roles in connecting innate and adaptive immune responses [16, 28]. The paracrine action of the endogenously produced prostanoids has been clearly documented in some immune organs and tissues. For example, cells of the thymus microenvironment, including those from the monocyte-macrophage lineage produce prostanoids, which contribute to the education of immature thymocytes. They also promote or block tolerance to self- and nonself-antigens. For example, tolerance to self might result from PGE2-driven apoptosis [54].

## 4. Prostanoid Receptor Signaling at the Interface between Innate and Adaptive Immunity

Receptors for prostanoids emerged as key regulators for both innate and adaptive immune responses, since they are widely expressed throughout the immune system, and function at multiple levels in connecting the innate and adaptive immunity [16, 24]. Biologically active prostanoids have a great impact on the phenotype and function of immune cells [27, 55]. Many accumulating data have reported that the production of inflammatory mediators and the expression of their receptors during the interaction of various immune cells markedly affect the outcome of immune responses in health and diseases [24, 33, 56]. Recognized as major prostanoids produced by immune cells, PGD2 and PGE2 have garnered much interest because they act as potent regulators of cell-cell interaction, antigen presentation, cytokine production, cytokine receptor expression, differentiation, survival, apoptosis, cell-surface molecule levels, and cell migration in both autocrine and paracrine manners. PGE2 can profoundly modulate various aspects of the immune and inflammatory responses [27, 33]. PGD2 has long been considered as a crucial regulator of allergic responses [57, 58]. However, it might exert many immunologically relevant anti-inflammatory functions in several experimental models [59–61]. In addition, it has been reported that mast cell-derived PGD2 acts as an antiangiogenic factor in expanding lung carcinoma [60]. Here, we focused on the immunobiology of PGD2 and PGE2 receptor signaling in tow examples of immune cell interactions: the first is the bidirectional activating crosstalk between DCs and NK cells and its crucial role in innate and adaptive immune regulations, the second is the physiological dialog between DCs and T cells during antigen presentation and the generation of adaptive immune responses in normal and pathological settings.

### 4.1. PGE2 and PGD2 Signaling during DC-NK Cell Crosstalk

Although various cell types participate in innate and adaptive immune regulations, DCs are a rather unique cell type, in that they may function in both innate and adaptive immunity, depending on their state of maturation and local microenvironmental conditions [62]. After their development from hematopoietic stem cells (HSCs) in the bone marrow, immature DCs migrate to the periphery and reside in nonlymphoid organs where they actively take up the antigens from the extracellular fluid [16]. In response to signal of inflammatory microenvironment, immature DCs undergo a process of maturation triggered by a variety of pathogen related molecules, such as bacterial LPS. They subsequently leave the tissues *via* the afferent lymphatic vessels, enter the draining lymph nodes in the T-cell-rich zone, present the processed antigens to naive T cells and induce a specific primary immune response [63]. DC maturation and migration involve soluble mediators, such as cytokines and prostanoids [24].

**Figure 1:**
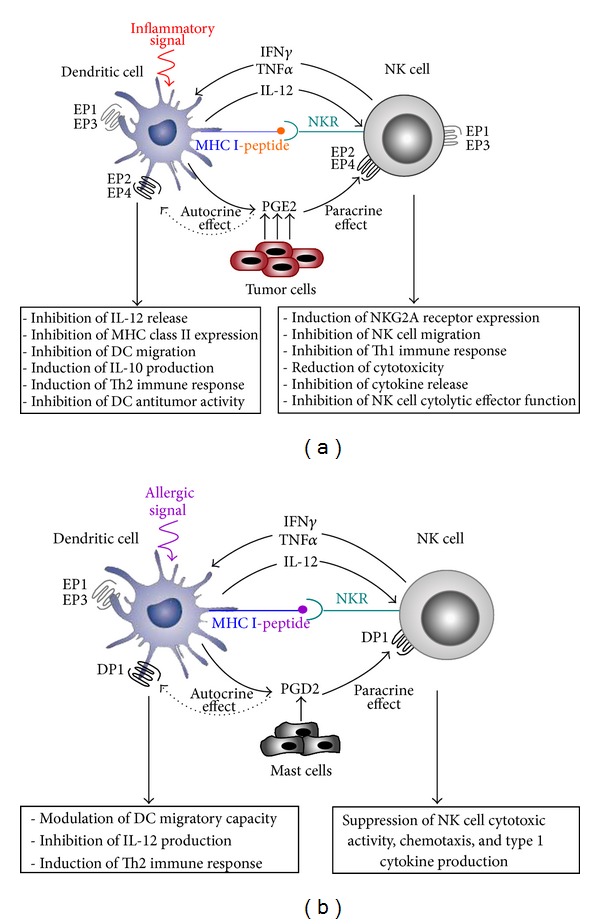
PGE2 and PGD2 receptor signaling in the bidirectional activating crosstalk between DCs and NK cells in normal and pathological conditions. (a) NK cells can interact with DCs through a range of cell surface receptors and production of various molecules both in the periphery and the secondary lymphoid organs. Activated immature DCs produce various cytokines, such as IL-12 that could act on NK cells recruited from the periphery by inflammatory signals and/or DC-derived chemokines. They induce NK-cell survival, proliferation, cytokine production, activation, and cytotoxicity. In turn activated NK cells produce cytokines, especially TNF*α*, which induces DC maturation process. When produced by DCs, PGE2 could inhibit NK cell activation through an EP2/EP4 receptor-dependent mechanism. Thus, inhibited NK cells could not stimulate DC maturation and function. The endogenously produced PGE2 can also reduce DC function in an autocrine manner *via* EP2/EP4 receptor subtypes. The tumor cells and stroma cells release diverse immunosuppressive agents, such as PGE2, which inhibits DC biology and NK effector functions through EP2 and/or EP4 receptor signaling. Tumor infiltrating DCs contribute also to increased levels of PGE2, which inhibits NK and DC functions and their crosstalk. (b) PGD2 produced by DCs or by mast cells has both autocrine and paracrine effects on DCs and NK cells through EP2 and/or EP4 receptors.

In addition to their originally and historically known function as the only professional APC capable of activating naïve T cells, DCs are also characterized with their potent ability to interact with innate immune cells, especially NK cells [64]. In fact, DCs and NK cells can interact with each other, and depending on the activation status of both cell types, this reciprocal dialog may lead to NK cell activation, DC activation, or apoptosis [65]. By modulating NK cell proliferation, IFN*γ* production [66], and cytotoxic activity [67], DCs play a pivotal role in orchestrating NK cell-mediated innate immune responses [68]. Conversely, NK cells can regulate the maturation, cytokine production, and immunostimulatory capacity of DCs [69]. The interaction between DCs and NK cells can be mediated through cell-cell contact, membrane-bound ligands, or by a variety of soluble factors, such cytokines and prostanoids that contribute to the modulation of the activity of both cell types. The reciprocal activating crosstalk between DCs and NK cells appeared to be markedly affected by PGE2 in normal and pathological settings [64]. Innate and effector functions of NK cells that require close interactions with activated DCs can be modulated by PGE2. In fact, NK cell functions (lysis, migration, proliferation, and cytokine production) are markedly influenced by DC-derived PGE2, which acts as a potent suppressor of DC-NK cell crosstalk (Figure 1(a)). During DC-NK cell crosstalk, DCs produce PGE2, which binds EP receptors expressed at the surface of NK cells [65] and suppresses their functions. Although NK cells express all PGE2 receptors [70], EP2 and/or EP4 receptor signaling appeared to play the main role in this effect [71, 72]. The suppression of DC-NK cell crosstalk by PGE2 can be mediated through the modulation of DC released chemokines and cytokines that are involved in NK cell recruitment [34, 73]. In some cases, defects in DC function resulted in the release of immunosuppressive factors, such PGE2 [6, 74, 75], which can induce the production of IL-10. This cytokine acts as a potent suppressor of the bidirectional activating crosstalk between NK cells and DCs [76]. Moreover, overproduction of immunosuppressive PGE2 has been clearly established in many cancers [7], causing reduced number of tumor-infiltrating DCs and a reduction in their APC function [77]. Defect in DC differentiation and APC function and cancer-associated immunodeficiency have been reported to be mediated by PGE2 EP2 receptor signaling [56].

Accumulating data reported that PGE2 EP2 and EP4 receptor signaling are potent immunoregulatory pathways affecting maturation, migration cytokine production, and Th cell-polarizing ability of DCs in health and diseases [70, 78, 79]. The general consensus is that EP1 and EP3 receptors have no role in DC biology as we and others have previously demonstrated [42, 78]. However, the recently published data by Singh et al. [80] showed that PGE2 affects Flt3L-dependent DC development from hematopoietic progenitor cells through EP1 and EP3 receptor-dependent mechanism, and DC generation was markedly lower in EP1 and/or EP3 knockout mice.

Although the most intensively studied PG member is PGE2, PGD2 emerged as a very interesting COX-2-derived metabolite involved in many biological processes [81, 82]. Described as a potent mediator with a key role in the development and pathogenesis of allergic diseases [83, 84], PGD2 can act as a potent regulator of immune and inflammatory responses [24, 27, 85, 86]. Within the immune system, PGD2 is generally recognized by its suppressive effects of cellular functions through DP1 receptor signaling, which is a powerful activator of the adenylate cyclase system and elevated levels of cAMP (Figure 1(b)). However, the stimulatory effects of PGD2 on type 2 cells are mediated by CRTH2 signal [47, 48]. Local production of PGD2 has been associated with impaired functions of NK cells in type 2 immune-mediated pathologies, such as asthma [87]. In fact, NK cell cytotoxic activity, chemotaxis, and type 1 cytokine production have been reported to be suppressed by PGD2 through DP1 receptor-dependent mechanism [88]. 

During the inflammatory response, PGD2 appears to exert a dual function with the capacity to act as either a mediator or a potent inhibitor of inflammation in some physiological or pathological conditions [59, 60]. This lipid mediator greatly affects DC migratory capacity and markedly downregulates the production of the Th1-promoting cytokine IL-12 through DP1, but not DP2 receptor signaling [86]. In superficial organs, such as skin and mucosa where DCs and mast cells are colocalized, PGD2 produced by mast cells can also suppress IL-12 release by DCs leading to Th2 polarized immune responses *in vivo* [89].

### 4.2. PGE2 and PGD2 Signaling during DC-T Cell Interaction

**Figure 2:**
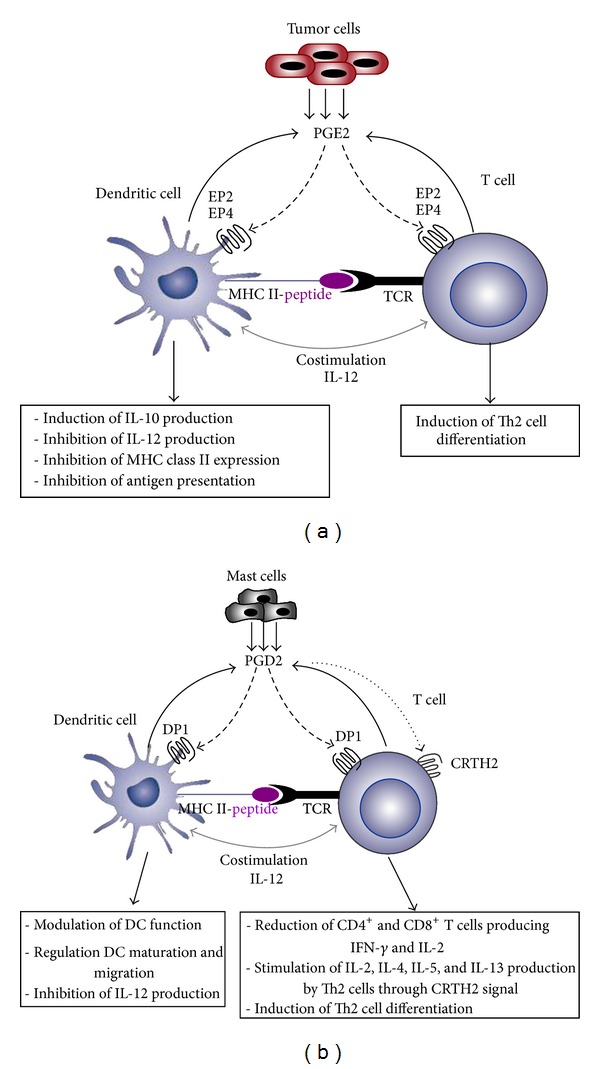
PGE2 and PGD2 receptor signaling during DC-T cell interaction in normal and pathological conditions. (a) During antigen presentation, PGE2 can be produced by DCs and acts on DC function and T cell differentiation through EP2/EP4 receptor signaling. Tumor cells are also an important source of PGE2, which acts as a potent suppressor of the APC function of DCs. (b) PGD2 produced by DCs and/or mast cells markedly affects T cell and DC functions *via* DP1 receptor signaling. PGD2 can also stimulate Th2 cell through CRTH2 signal.

Activation of naïve T cells and their polarization into effector cells requires close interactions with mature DCs in the lymphoid organs. During the maturation process and/or during DC/T lymphocyte contact, many factors including cytokines and inflammatory lipid mediators, such as PGE2 and PGD2, can be produced (Figure 2(a)). Cytokine production and antigen presentation by DCs are inhibited by PGE2 [15] through EP2 and/or EP4 receptors signaling [42, 90]. PGE2 is also known to induce many immunosuppressive factors, such as indoleamine 2, 3-dioxygenase (IDO) [91, 92], which can suppress T-cell proliferation and survival and induces immunological tolerance [93]. The immunomodulatory effects of PGE2 can be prolonged and sustained by these immunosuppressive factors known by their ability to induce a tolerogenic type of DCs [15, 91]. Moreover, it has been reported that DCs, exposed to autocrine and/or paracrine PGE2, induce the differentiation of naive T cells into Th2 cells, which produce high levels of IL-4 and no IFN-*γ* [94]. 

Another COX metabolite that can be released during DC-T cell interaction is PGD2 (Figure 2(b)). In the lymphoid organs, the local production of PGD2 by DCs and/or T cells [95, 96] may affect the immune response by targeting Th1-driving cytokine IL-12 [27], suggesting a key role of PGD2 in the outcome of the adaptive immunity. PGD2 can greatly affect the migratory properties of DCs. Gosset et al. [86] reported that PGD2 and its metabolite 15d-PGJ2 are able to block the maturation of human monocyte-derived DCs (MD-DCs) in a DP receptor-dependent manner. Paracrine effects of PGD2 on DCs and T cells have also been shown [97]. In fact, some immune cells, such as macrophages, might secrete PGD2, which suppresses DC migration and T cell activation. By blocking DC migration, PGD2 prevents further immune stimulation, thus contributing to the resolution of inflammation.

## 5. Epigenetic Modifications of Prostanoids: Fine Regulations with More Complexity 

The potential relevance of prostanoid signaling in inflammation, cancer, or disease susceptibility and individual variations in drug responses will be an important area for investigation. The study of prostanoid involvement in connecting innate and adaptive immunity is a complex process. In addition to the environmental factors and the genetic background to diseases, including cancer and asthma, epigenetic mechanisms involved in the fine regulation of prostanoid biosynthesis and receptor signaling appeared to be crucial in controlling the different components of the COX pathways [98, 99]. In fact, genes encoding for inflammatory prostanoids and their receptors are subjected to epigenetic modifications by acetylation of core histone. Immune disorders, such as asthma and cancer, are characterized by the expression of various inflammatory genes that can be epigenetically regulated by acetylation of core histone. For example, increased activity of histone acetyltransferase and reduced activity of histone deacetylase have been observed in asthmatic patients [100]. Histone acetylation was also found to be a critical regulator of EP expression in cancer [99]. The epigenetic downregulation of the *EP2* gene by DNA CpG methylation was observed to be associated with progression of neuroblastomas [101]. Together, these data suggest that prostanoids and their receptors can be functionally regulated epigenetically, and the epigenetic mechanisms controlling the different components of prostanoid biosynthesis pathway and signaling should be considered in the development of therapeutic approaches aimed at targeting prostanoid biosynthesis and signaling in immune disorders. 

## 6. Concluding Remarks 

The molecular and cellular basis of the immune regulation by prostanoids in physiological and pathological situations remains a topic of great interest. Receptors for major prostanoids, especially PGD2 and PGE2, are widely expressed throughout the immune system, and function at multiple levels in connecting the innate and adaptive immunity. PGD2 and PGE2 receptor signaling emerged as key regulators for both innate and adaptive immune response. Both DC-T cell interactions and DC-NK cell reciprocal activating crosstalk provide a target for pharmacological interventions in normal and pathological settings. The manipulation of the local cellular prostanoids and their receptors expressed by innate and adaptive immune cells during their functional crosstalk might be an interesting approach to modulate DC and/or NK cell functions for specific immune responses, especially in cancer, asthma and inflammation.
